# Autophagy, the innate immune response and cancer

**DOI:** 10.1002/1878-0261.12774

**Published:** 2020-08-30

**Authors:** Chelsea Gerada, Kevin M. Ryan

**Affiliations:** ^1^ Cancer Research UK Beatson Institute Garscube Estate Glasgow UK; ^2^ Institute of Cancer Sciences University of Glasgow Garscube Estate Glasgow UK

**Keywords:** autophagy, cancer, immunotherapy, innate immune response, tumour microenvironment

## Abstract

Autophagy is a cellular degradation and recycling system, which can interact with components of innate immune signalling pathways to enhance pathogen clearance, in both immune and nonimmune cells. Whilst this interaction is often beneficial for pathogen clearance, it can have varying outcomes in regard to tumorigenesis. Autophagy and the innate immune response can have both pro‐ and antitumorigenic effects at different stages of tumorigenesis due to the plastic nature of the tumour microenvironment (TME). Although both of these components have been studied in isolation as potential therapeutic targets, there has been less research concerning the interaction between autophagy and the innate immune response within the TME. As the innate immune response is critical for the formation of an effective antitumour adaptive immune response, targeting autophagy pathways in both tumour cells and innate immune cells could enhance tumour clearance. Within tumour cells, autophagy pathways are intertwined with pattern recognition receptor (PRR), inflammatory and cell death pathways, and therefore can alter the immunogenicity of the TME and development of the antitumour immune response. In innate immune cells, autophagy components can have autophagy‐independent roles in functional pathways, and therefore could be valuable targets for enhancing immune cell function in the TME and immunotherapy. This review highlights the individual importance of autophagy and the innate immune response to tumorigenesis, and also explains the complex interactions between these pathways in the TME.

## Graphical abstract

Both autophagy, a cellular recycling process, and the innate immune response can have different effects on tumour formation at different stages. Interestingly, autophagy and the innate immune response interact during tumorigenesis to have both tumour‐promoting and tumour‐inhibiting affects. By dissecting the interaction between autophagy and the innate immune response during tumour formation, novel therapeutic strategies may be identified.

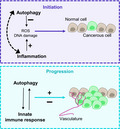

**Abbreviations**
AMPK: 5' adenosine monophosphate‐activated protein kinase
ATG: autophagy‐related gene
BECN1: beclin‐1 gene
CCL: chemokine (C‐C motif) ligand 5
CD: cluster of differentiation
cDC: classical dendritic cell
cGAMP: cyclic GMP‐AMP
cGAS: cyclic GMP‐AMP synthase
CIS: cytokine‐inducible SH2‐containing protein
CRC: colorectal cancer
DAMP: damage‐associated molecular pattern
DC: dendritic cell
ERK: extracellular signal‐regulated kinase
FRA1: fos‐related antigen 1
HCC: hepatocellular carcinoma
HMBG1: High‐mobility group box 1
IFN: Interferon
IL: Interleukin
ILC: innate lymphoid cell
ISG: interferon‐stimulated gene
KRAS: kirsten rat sarcoma viral oncogene homolog
LAMP: lysosomal‐associated membrane protein 1
LC3: microtubule‐associated protein 1A/1B‐light chain 3
LPS: Lipopolysaccharide
MAP3K7: mitogen‐activated protein kinase kinase kinase 7
MHC: major histocompatibility complex
MoDC: monocyte‐derived dendritic cell
MSDC: myeloid‐derived suppressor cell
mTOR: mammalian target of rapamycin
NF‐κB: nuclear factor kappa‐light‐chain‐enhancer of activated B cells
NK: natural killer cell
NLR: nod‐like receptor
NLRP: NOD‐, LRR‐ and pyrin domain‐containing protein
PAMP: pattern‐associated molecular pattern
PDAC: pancreatic ductal adenocarcinoma
pDC: plasmacytoid dendritic cell
PI3KC3: class III phosphatidylinositol 3‐kinase
PRR: pattern recognition receptor
PTEN: phosphatase and tensin homolog
RAGE: receptor for advanced glycation end products
RANKL: receptor activator of nuclear factor kappa‐Β ligand
RIPK: receptor‐interacting serine/threonine‐protein kinase
ROS: reactive oxygen species
SQSTM1: sequestosome 1
STAT: signal transducer and activator of transcription
STING: stimulator of interferon genes
TAM: tumour‐associated macrophage
TBK1: TANK‐binding kinase 1
TFEB: transcription factor EB
TGF‐β: transforming growth factor beta
Th: T‐helper cell
TIGIT: T‐cell immunoreceptor with Ig and ITIM domains
TIM‐4: T‐cell membrane protein 4
TLR: Toll‐like receptor
TME: tumour microenvironment
TRAIL: TNF‐related apoptosis‐inducing ligand
TRIF: TIR domain‐containing adapter‐inducing interferon‐β
UVRAG: UV radiation resistance‐associated gene
WIPI2: WD repeat domain phosphoinositide‐interacting protein 2

## 1 Introduction

Autophagy serves as a degradation system to ensure cellular homeostasis by regulating the turnover and/or removal of cellular components such as proteins and organelles. Autophagy is initiated in response to environmental perturbations and interacts with metabolic, cell death and innate immune pathways to ensure appropriate cellular responses to these perturbations [1].There are three primary forms of autophagy, which are morphologically unique, but ultimately result in cargo degradation and recycling via the delivery to the lysosome [1]. In microautophagy, cytoplasmic cargo is engulfed via invagination of lysosomal or endosomal membranes [2]. Macroautophagy involves autophagy adaptors, which tag cellular cargo so it can be recognised by double‐membrane autophagosomes, which ultimately fuse with lysosomes [1]. Chaperone‐mediated autophagy is different again. Cargo proteins containing a KFERQ‐like motif bind to chaperones enabling transport directly across the lysosomal membrane via LAMP2a [3]. Either via direct transport to the lysosome or autophagosome–lysosome fusion, cargo is degraded by lysosomal hydrolases.

Similar to autophagy, the immune response serves to detect changes such as cellular damage or infection, to ensure homeostasis. The innate immune response involves the detection of cellular abnormalities, which can cause cell death and/or the release of cytokines and chemokines, which in turn activate surrounding innate immune cells. This occurs via damage‐associated molecular pattern (DAMP) and pattern‐associated molecular pattern (PAMP) sensing by pattern recognition receptors (PRRs), which can activate cell death pathways and the production of cytokines and chemokines [4]. Cytokines and chemokine release cause the activation of tissue‐resident innate immune cells and the influx of blood‐derived innate immune cells, respectively. Innate immune cells include macrophages and dendritic cells (DCs) from the myeloid lineage and cells from the lymphoid lineage such as innate lymphoid cells (ILCs) and natural killer cells (NK cells). The various cell innate cell subsets and function will be reviewed later on in this article. The innate immune response is critical for the formation of an effective adaptive immune response as antigen‐presenting cells travel to lymph nodes to activate particular T‐helper cell subsets, which drive adaptive immunity [5].

Both autophagy and the innate immune response not only play dual roles in different stages of tumorigenesis but also can interact with each other to form either a pro‐ or antitumorigenic response. Therefore, increasing understanding of how autophagy and the innate immune response interact in the context of tumorigenesis may provide novel therapeutic targets to enhance antitumour immunity and immunotherapy. This review aims to overview the role of autophagy and the innate immune response in tumorigenesis individually and dissect how these components influence each other to either promote or inhibit tumorigenesis.

## 2 Autophagy: dual roles in cancer development

**Fig. 1:**
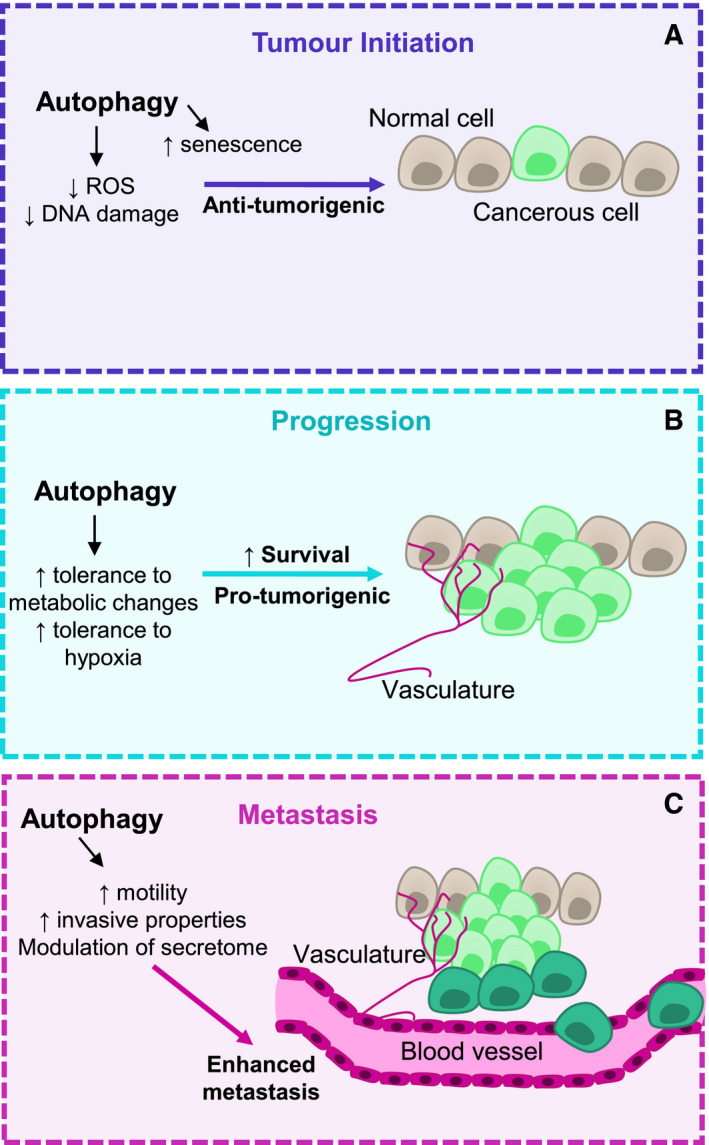
Role of autophagy at different stages of tumorigenesis. Autophagy has different effects at different stages of tumorigenesis. In tumour initiation (A), autophagy limits the production of DNA‐damaging agents such as ROS in response to cellular stress. Additionally, autophagy has been shown to promote several aspects of senescence in tumour cells, which can result in decreased tumour growth. During tumour growth, autophagy enhances tumour cell survival via increasing resistance to metabolic changes and hypoxia within the tumour microenvironment (B). Autophagy can also enhance tumour cell metastasis via interacting with pathways involved in cell motility and invasion (C). Additionally, autophagy can modify the secretome in the tumour microenvironment to promote invasion into the vasculature and establishment at distal sites.

It is critical for cells to adapt to environmental perturbations to ensure that homeostasis and the overall health of the organism are maintained. Autophagy, a complex recycling pathway that involves the turnover of cellular components, is central to this process. It has previously been demonstrated that by maintaining cellular homeostasis, autophagy can be tumour‐suppressive [6]. However, in established tumours, autophagy can compensate for a plethora of environmental changes such as hypoxia, altered metabolic programming, nutrient starvation and cell stress, to protect cells from death [7]. Therefore, it is critical to dissect the role of autophagy at different stages of tumour development. Most studies investigating the role of autophagy in tumorigenesis tend to focus on macroautophagy, and as such, this review primarily focuses on macroautophagy (which we refer to hereafter more simply as autophagy). However, it is important to note that chaperone‐mediated autophagy plays a role in the development of tumorigenesis, whilst the role of microautophagy is less clear [8]. In the prevention of solid tumour development or in the very early phases of solid tumour formation, autophagy has been suggested to be tumour‐suppressive [6, 9] (Fig. 1). This is supported by evidence in which mutations that activate mTORC1 and subsequently inhibit autophagy are common in cancer, suggesting that inhibition of autophagy during early stages of tumour progression is favourable, or at least permissible [10, 11]. Genetic studies of autophagy components *in vivo* have shown that defects in autophagy can cause an increase in tumour initiation [12, 13, 14]. This increased tumour initiation has been linked to increases in reactive oxygen species (ROS), DNA damage and dysfunctional mitochondria [15]. Additionally, autophagy is required for the establishment of cellular senescence in response to oncogenic stress [16, 17, 18]. Senescence can prevent malignant transformation, however can be detrimental overall, due to factors such as potential neoplastic conversion [17].

Interestingly, the balance of autophagy’s effects is considered to be more protumorigenic in later stages of tumorigenesis [19] (Fig. 1). This is demonstrated in a mouse lung cancer model where *Atg7* deletion, which causes a block in autophagy, initially accelerated tumour growth, and however at later stages caused a decrease in tumour burden and ultimately an increase in survival [13]. There is also evidence that autophagy plays a role in promoting tumour initiation in the context of *Apc* ^+/‐^ mice [20]. In these mice, conditional inactivation of ATG7 in intestinal epithelial cells inhibited the formation of precancerous lesions by impairing metabolic growth and increasing the immune response linked with gut microbiota [20]. The ability of autophagy to promote tumorigenesis has been linked with changes in metabolism and hypoxia, which increases stress tolerance [19].

Autophagy also supports tumour metastasis, a multistep process that involves tumour cells undergoing an epithelial‐to‐mesenchymal transition (EMT). EMT allows the tumour cells to escape into the vasculature and eventually extravasate and grow at another site. Autophagy aids in this process by turnover of cell migratory machinery, extracellular matrix proteins and modulation of the secretome, which ultimately enhances cell mobility and invasiveness. The mechanistic basis for the role of autophagy in metastasis has been reviewed elsewhere [7]. The factors that dictate whether autophagy is pro‐ or antitumorigenic are complex; however, it is important to elucidate these factors to determine whether targeting autophagy for cancer therapy is a viable strategy. The genetic background of the tumour may determine the pro‐ or antitumorigenic potential of autophagy. p53 is a tumour suppressor whose expression is often altered in cancer [21]. In a humanised mouse model of pancreatic ductal adenocarcinoma (PDAC), p53 expression altered the effect of autophagy loss (*Atg5* or *Atg7* deletion) in mice, which expressed the activated oncogenic allele of *Kras* in the pancreas [22]. Autophagy loss in mice lacking p53 caused an increase in precursor lesion formation and accelerated tumour onset, whereas autophagy loss in mice with wild‐type p53 caused a block in PDAC development [22]. In a different PDAC model driven by mutant *Kras* with a loss of the tumour suppressor *Pten*, deletion of *Atg7* did not block PDAC formation when *Pten* was hemizygous and animals died earlier in comparison with autophagy‐competent animals [23]. When both alleles of *Pten* were deleted, autophagy‐deficient tumours were formed; however, loss of *Atg7* did not accelerate tumour onset. This may be due to the rapid onset of tumours when *Pten* is completely lost. Together, this demonstrates that autophagy loss can also promote tumour development in a *Pten*‐deficient background. It is important to note that once a tumour has been established, the genetic status *of p53* or *Pten* may not determine whether autophagy has an antitumorigenic role due to a variety of other factors involved in the crosstalk between tumorigenesis and autophagy. Other factors that have been linked to the dual role of autophagy in tumorigenesis include crosstalk with cell death pathways, modulation of antitumour immune responses and controlling homeostasis of proteins and organelles [24]. For the purposes of this review, we will focus on the interplay between autophagy and the innate immune response in the context of tumorigenesis.

## 3 The dual role of the innate immune response in cancer development

Similarly to autophagy, the innate immune response also plays a complex role in tumorigenesis. The innate immune response is critical in sensing malignant cells and moulding an effective adaptive immune response. However, components of the innate immune response can promote tumour formation and can contribute to rendering tumours immunologically silent. It is important to identify the factors driving the pro‐ and antitumorigenic effects of the innate immune response to increase the efficacy of immunotherapy and to identify novel therapeutic targets.

### 3.1 A positive feedback loop between inflammation and tumour initiation

**Fig. 2:**
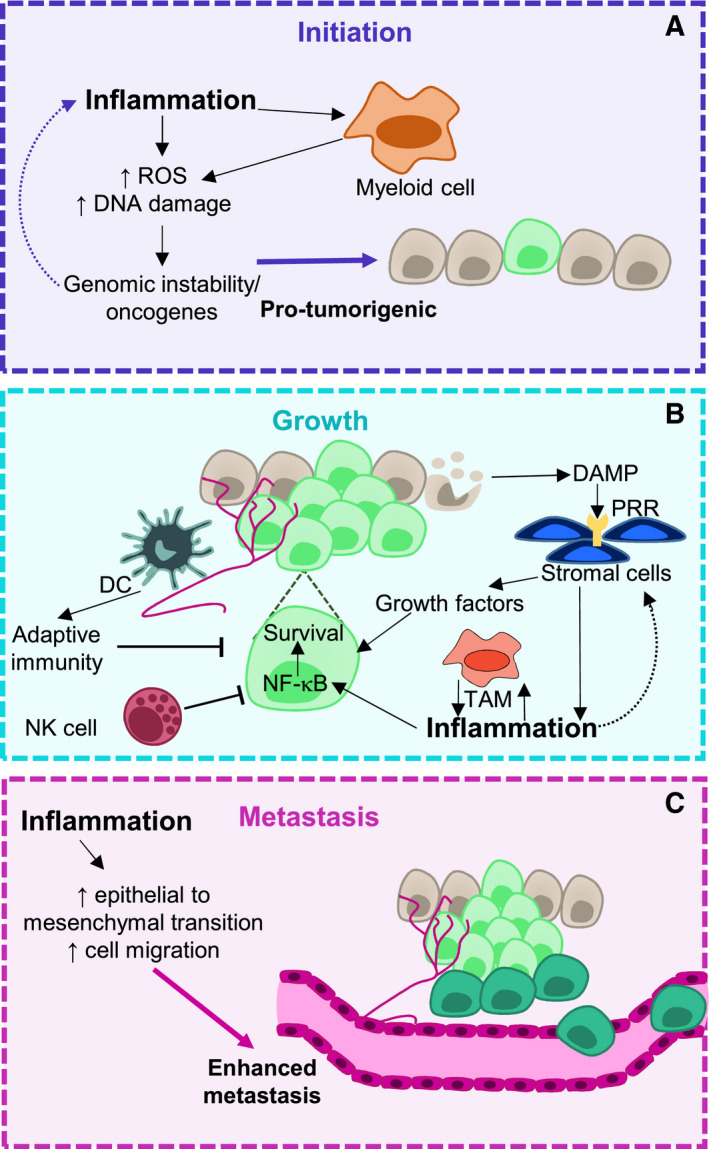
Role of the innate immune response at different stages of tumorigenesis. Chronic inflammation can stimulate tumour initiation via the production of DNA‐damaging agents such as ROS (A). Additionally, certain oncogenes can feedback into this process by potentiating pathways in tumour cells. Myeloid cells have been shown to contribute to this process via the generation of DNA‐damaging agents. During tumour growth, tumour cells release DAMPs into the tumour microenvironment (B). Damage‐associated molecular patterns (DAMPs) can be sensed by pattern recognition receptors (PRRs) on stromal cells, causing these cells to release growth factors and inflammatory cytokines, which can promote tumour survival. Inflammatory cytokines activate NFκB in tumour cells, which can stimulate cell survival pathways. The inflammatory microenvironment can stimulate innate immune cells such as tumour‐associated macrophages (TAMs), which can feed back into the inflammatory microenvironment, whereas other innate immune cells play an antitumorigenic role such as natural killer (NK) cells and dendritic cells (DCs). The innate immune response also enhances metastasis via inflammation, which can increase epithelial‐to‐mesenchymal transition and cell migration (C).

Inflammation driven by the innate immune response has been linked with the initiation of certain cancers. Many lifestyle factors linked to cancer development, such as smoking, alcohol consumption or a high‐fat diet, have also been shown to increase inflammation [25, 26, 27]. Additionally, chronic inflammatory conditions, such as inflammatory bowel disease, can render patients more susceptible to developing cancer [28, 29]. The proposed mechanism behind this association is that chronic inflammation drives a mutagenic environment [30]. Inflammatory mediators such as ROS can cause DNA damage and genomic instability [31] (Fig. 2). This has been demonstrated in the intestine, where chronic inflammation causes an accumulation of mutations in *TP53* and other oncogenes in epithelial cells [31, 32, 33].

Whilst chronic inflammation can result in the accumulation of mutations in oncogenes and tumour suppressors, certain mutations can also potentiate the inflammatory response. For example, mutations in *TP53* can alter NF‐κB regulation, resulting in an increase in NF‐κB‐dependent inflammatory gene expression [34, 35]. Additionally, expression of oncogenes KRAS and c‐MYC can enhance inflammatory cytokine and chemokine production [36]. Inflammation can also contribute to tumour initiation via contributing to the formation of cancer stem cells. Cancer stem cells have the capacity to self‐renew and differentiate and thus can sustain tumour growth [37]. In an irradiated glioblastoma model, NF‐κB signalling in glioblastoma cells caused the recruitment of Ly6G + inflammatory cells, which facilitated the conversion of glioblastoma cells to glioblastoma stem cells [38]. In tumours, Ly6G + inflammatory cells can include granulocytic myeloid‐derived suppressor cells and tumour‐associated neutrophils, which can secrete factors to promote tumour growth [39, 40]. Additionally, the IL‐6/STAT3 signalling axis has been shown to play a role in colorectal cancer cell stemness via FRA1 deacetylation [41]. Overall, it is clear that inflammation can drive oncogenic events in cancer initiation and these oncogenic events can potentiate the inflammatory response.

### 3.2 Pattern recognition receptors contribute to inflammation in the TME

The DAMPs are produced during tumorigenesis due to hypoxia, changes in cell metabolism and cell death. PRRs can sense DAMPs, amplify inflammatory signalling pathways and play a critical role in innate immune cell activation. Toll‐like receptors (TLRs) recognise PAMPs and DAMPs in endosomal and plasma membranes and signal through adaptor proteins to activate NF‐κB signalling (Fig. 2). Like most other aspects of the innate immune response, TLRs can have both a pro‐ and antitumorigenic effect [42]. In a *Tlr3, Tlr7* and *Tlr9* knockout mouse model, transplantable tumour cells were rejected after 10 days and this was associated with differences in inflammation and an effective CD8^+^ T‐cell response, suggesting that TLR signalling in non‐malignant cells may regulate antitumour immune responses [43]. TLRs in the plasma membrane, such as TLR4, mainly recognise pathogen cell wall components and thus can link the microbiome to cancer development. In hepatocellular carcinoma (HCC), TLR4 and intestinal microbiota were required for tumour promotion and this was mediated by resident liver cells [44]. Alternatively, TLR signalling through innate immune cells can have antitumour effects via decreasing immunotolerance [42].

Nod‐like receptors (NLRs) are cytosolic PRRs, which can contribute to the formation of the inflammasome and the production of IL‐1β and IL‐18, as well as transcription and NF‐κB pathway regulation [45]. The various associations and roles of NLRs in cancer have been reviewed elsewhere [46]; however, it is important to highlight that there are many members of the NLR family that all have distinctive functions in tumorigenesis. For example, activating mutations in NLRP1, a critical inflammasome sensor in the skin, can cause skin hyperplasia and an increased disposition to skin cancer development [47], whereas NLRP12 can regulate noncanonical NF‐κB activation as a checkpoint of tumorigenesis in the context of colitis‐associated colon cancer [48].

DNA from cancer cells can activate the cGAS/ STING pathway to cause the induction of the type I interferon (IFN) response. Cytosolic self‐DNA in tumours can activate this pathway to cause the induction of cellular senescence. Extracellular DNA released into the TME can stimulate this pathway in innate immune cells to promote immunosurveillance; therefore, perturbation of the cGAS/ STING pathway can cause tumour progression [49].

Ultimately, it is clear that the sensing of PAMPs and DAMPs by PRRs within tumour cells and cells in the TME can initiate inflammatory signalling. The resulting innate immune responses can stimulate or impede tumour development.

### 3.3 Inflammation enhances tumorigenesis

Once an inflammatory response has been initiated during tumorigenesis, components of this response can support tumour growth and metastasis (Fig. 2). Inflammatory cytokines can signal through cytokine receptors on transformed cells to increase prosurvival NF‐κB‐dependent and NF‐κB‐independent pathways to increase the survival and proliferation of malignant clones [30, 50]. Additionally, many proinflammatory cytokines such as IL‐6 can increase tumour cell proliferation [51]. Inflammation not only drives tumour cell survival and proliferation, but also drives the formation of the TME. This occurs through stimulation of fibroblasts and stromal cells, which perpetuate inflammation and secretion of factors needed for angiogenesis and tumour growth [52]. The ability of tumours to metastasise greatly contributes to cancer‐related deaths. Inflammation has been shown to play roles in many stages of metastasis such as epithelial‐to‐mesenchymal transition, intravasation and extravasation and cell migration; however, this has been reviewed elsewhere [30].

### 3.4 Myeloid cells

Innate immune cells, particularly myeloid cells, help orchestrate the inflammatory response throughout tumorigenesis mainly via the production of pro‐ and anti‐inflammatory cytokines/chemokines. In a model of intestinal tumorigenesis, myeloid cell‐derived H_2_O_2_ caused genome‐wide DNA mutations in epithelial cells, which caused invasive growth [31]. Additionally, in the absence of carcinogen challenge, ROS produced by myeloid cells caused tumour initiation [31], indicating that myeloid cells can contribute to cancer initiation and progression. DCs and macrophages derive from the myeloid cell lineage, and both have complex roles in tumorigenesis due to a high degree of functional plasticity.

Macrophages, depending on their environment, origin and tissue residence, can aid in tissue regeneration and repair or contribute to inflammation and killing of damaged cells [53]. Tumour‐associated macrophages (TAMs) are often the most abundant immune cell in the TME and are derived from both tissue‐resident macrophages and monocyte‐derived macrophages [54] (Fig. 2). In endometrial and breast cancers, TAMs have a transcriptional programme distinct from monocytes and their respective tissue‐resident macrophages, which is associated with patient survival [55], displaying that the TME can drive changes in TAMs during tumorigenesis. In addition to different developmental origins, TAMs have further functional heterogeneity. Whilst inflammatory macrophages have the capacity to be antitumorigenic through promotion of Th1 T‐cell and NK cell responses, TAMs are generally associated with having a protumorigenic phenotype [56]. This is due to functional changes, which include a highly activated arginase pathway, high phagocytic activity, high levels of expression of mannose and galactose receptors and ultimately the promotion of a Th2 T‐cell response [53]. Overall, these factors contribute to an ineffective antitumour immune response. Another factor that affects TAM functionality is the spatial location of TAM within the tumour. At the leading edge of tumours, TAMs can promote tumour cell invasion and can secrete molecules, which promote matrix remodelling to promote metastasis [55, 57]. Overall, TAM diversity in the tumour landscape changes over time due to different environmental pressures, resulting in different outcomes for tumorigenesis. Therapeutically, understanding what causes TAMs to become protumorigenic and whether it is possible to re‐educate TAMs to adopt an antitumorigenic phenotype is a key area of investigation.

DCs localise to tissues, where the local microenvironment influences their functionality [54]. As highly effective antigen‐presenting cells (APCs), DCs can take up antigens and present these antigens on MHC/HLA molecules to T cells, to initiate the adaptive immune response [54]. Classical dendritic cells (cDCs) are critical in forming and directing an effective antitumour immune response through functions in lymphoid tissue and in the tumour itself (Fig. 2). This is demonstrated in a recent study of spontaneous, oncogene‐driven models of pancreatic and lung cancer. In PDAC, a lack of cDC infiltration was associated with a tumour‐promoting Th17 response and a poor antigen‐specific CD8 T‐cell response, whilst in lung adenocarcinoma, abundant functional cDCs were associated with a Th1‐dominant response with antigen‐specific CD8 T cells [58].

Other subsets of DCs include monocytic DCs (MoDCs) and plasmacytoid DCs (pDCs). pDCs express specific TLRs and produce large amounts of type I IFN when activated by DAMPs. Whilst pDCs can have antitumorigenic roles, in some circumstances pDCs can become protumorigenic. This can be identified in melanoma where pDCs have been associated with poor clinical outcomes and have been shown to have distinctive features in the TME, such as promoting Th2 responses and regulatory immune profiles [59]. MoDCs originate from monocytes during inflammation and are efficient in the uptake and processing of antigens. In some models, MoDCs have been associated with CD8 T‐cell infiltration; however, other models display an immunosuppressive phenotype [54]. Collectively, DCs have functions that are critical to an effective antitumour immune response; however, due to their plasticity they can develop immunosuppressive functions and promote ineffective immune responses in the context of cancer. Similar to macrophages, much current research is being focused on methods of improving antitumorigenic functioning of DCs in cancer [60].

### 3.5 Innate lymphoid cells

Another innate immune cell type that plays a critical role in the development of the TME is innate lymphoid cells (ILCs). Whilst ILCs are derived from lymphoid progenitors, they do not express antigen‐specific B‐cell or T‐cell receptors. Four major groups of ILCs have been defined: natural killer (NK) cells, group 1 ILCs (ILC1s), group 2 ILCs (ILC2s) and group 3 ILCs (ILC3s) [61]. NK cells have cytotoxic potential against tumour cells and can target cells that lack MHCI expression and express the correct balance of activating and inhibitory ligands to NK cell receptors. ILC1s, ILC2s and ILC3s possess properties of Th1, Th2 and Th17 cells, respectively, and therefore help direct the innate and adaptive immune response [61].

Whilst NK cells have antitumour activity *in vitro*, less is known about their infiltration in solid tumours in a human setting. Patients with solid tumours have lower natural cytotoxic activity in peripheral blood compared with healthy controls, and poor NK cell function has been correlated with metastasis development [62]. In some cancers, such as renal cell cancer, NK cell infiltration is associated with more favourable outcomes [63], whereas in others, such as non‐small‐cell lung cancer, NK cell infiltration does not seem to have an impact [64]. This is due to differences in NK cell functionality in different TMEs. Tumours have been shown to decrease both NK cell infiltration and functionality through the expression of cytokines and chemokines. Therefore, targeting certain aspects of the tumour environment could increase NK cell killing of tumour cells [62]. Similar to T cells, NK cells can also express immune checkpoint molecules, which modulate their function in tumours. CIS (encoded by the *Cish* gene in mice) is a checkpoint of NK cell activity in tumour immunity as it negatively regulates IL‐15 signalling in NK cells [65]. *Cish* knockout mice were resistant to melanoma, prostate and breast cancer metastasis in an NK cell‐dependent manner [65]. TIGIT is another recently identified NK cell checkpoint and has been shown to be associated with NK cell exhaustion in mice and patients with colon cancer [66]. Blocking TIGIT in different mouse models caused a decrease in NK cell exhaustion and an NK cell‐dependent increase in tumour‐specific T‐cell immunity [66]. Overall, this demonstrates that improving NK cell function in tumours may also aid in promoting an effective T‐cell response and therefore is a critical line of study for checkpoint immunotherapy.

ILC1s, ILC2s and ILC3s are more recently discovered innate immune cell types; therefore, less is known about their presence and role in solid tumours. In colorectal and lung tumours, NK cells were the predominant ILC; however, the other subtypes were present at different abundances [67]. ILC1s are typically activated by cytokines such as IL‐12, IL‐15, IL‐18 and TGF‐β, and their function varies depending on the cytokines present [61]. In mice, IL‐15‐rich environments cause an expansion of tissue‐resident ILC1‐like cells, which can limit tumour growth independent of NK cells [68]. In tumours with high TGF‐β expression, ILC1s have minimal effect or increase tumour growth. In mice, TGF‐β caused the conversion of NK cells intro ILC1s, which caused higher tumour growth and metastasis [69].

ILC2s appear to contribute to an immunosuppressive environment in gastric cancer [70], and in mouse models of breast and liver cancer, they appear to be protumorigenic [71]. However, in a mouse model of lung metastatic melanoma secretion of IL‐5 by ILC2‐like cells drove an increase in eosinophil recruitment and decreased tumour growth [72]. Recently, ILC2s have been shown to infiltrate PDACs, where they are activated by IL‐33 to restrict pancreas‐specific tumour growth [73]. Furthermore, PDAC‐infiltrating ILC2s expressed PD‐1, and PD‐1 blockade caused an expansion of ILC2s to increase tumour control [73].

ILC3s are mainly found in mucosal tissues and can produce cytokines such as IL‐17 and IL‐22 [61]. In a bacterial model of colorectal cancer (CRC), ILC3s promote tumour growth via IL‐17 and IL‐22 secretion [74]. However, in nonintestinal tumours there is evidence for ILC3s being protumorigenic. For example, in human breast cancer ILC3s correlated with an increased likelihood of lymph node metastasis [75]. In a mouse model of breast cancer, ILC3s interacted with stromal cells to increase RANKL, a cancer cell motile factor [75]. Overall, plasticity of ILCs in different TMEs and the factors that govern this functional plasticity could be utilised to enhance antitumour immune responses and is an important area of future research.

The innate immune response has complex interactions with the TME, both driving its establishment and being influenced by it. Components of the innate immune response have the capacity to be both anti‐ and protumorigenic depending on environmental factors such as inflammation, and cytokine and chemokine expression. Uncovering factors that shift the functional properties of innate immune cells towards being antitumorigenic will be critical for the generation of an effective antitumour immune response and to increase the success of immunotherapy. One factor that has a key role in influencing both the immunogenicity of tumours and the functions of innate immune cells is autophagy.

## 4 **Autophagy as a key regulator of innate immunity in cancer**

Damaged or malfunctioning cells must be sensed to generate inflammation, stimulate surrounding innate immune cells and cause infiltration by circulating immune cells [5]. This ultimately leads to the removal of damaged cells through a coordinated innate and adaptive immune response. However, both the immune cells and the local microenvironment must be regulated to ensure the most effective response is generated without causing excessive tissue damage. At the molecular level, autophagy interacts with inflammatory, antigen presentation and innate immune signalling pathways and is influenced by the environmental context of the cell, allowing for components of innate immunity to be situationally regulated [76, 77]. During tumorigenesis, the formation of the TME is modulated by autophagy [6] and this unique microenvironment causes changes in autophagy signalling pathways in tumour cells, stromal cells and innate immune cells. Therefore, there is potential to harness autophagy to enhance the antitumour innate immune response and increase the effectiveness of immunotherapy. It is important to dissect how autophagy modulates innate immune signalling pathways in tumour cells and innate immune cell function within the TME, in order to identify novel therapeutic targets.

### 4.1 Autophagy and the regulation of innate immunity in tumour cells and stromal cells

Tumour cells and surrounding supporting cells have the potential to respond to DAMPs/ PAMPs in the TME to generate an inflammatory response. Autophagy is intrinsically connected to DAMP release, PRRs and downstream signalling pathways and can therefore influence the intrinsic innate immune response and the formation of the TME. Dissecting the relationship between these components in different cancer models at different stages of tumorigenesis is critical to understanding whether these relationships are pro‐ or antitumorigenic, and whether they can be exploited to enhance cancer therapies.

### 4.2 Autophagy regulates DAMP release

Damaged cells can release cellular components such as ATP, nucleic acids, HGMB1 and IL‐1B into the extracellular space [78]. These components act as DAMPs and can be sensed by cells in the microenvironment to activate the innate immune response [78]. Autophagy regulates the release of many of the aforementioned DAMPs. For example, autophagy can promote ATP secretion before cell death through trafficking ATP to the cell membrane via the endolysosomal pathway [79]. In immunocompetent mice, autophagy is required for ATP release from tumour cells in response to radiotherapy, which causes a more effective antitumour immune response [80]. HMBG1, a chromatin nuclear‐associated protein, is released from dying tumour cells and acts as a DAMP and a regulator of autophagy. Extracellular HMGB1 can bind to RAGE to inhibit mTOR and increase autophagy whilst limiting apoptosis via a p53‐dependent mitochondrial pathway in PDAC [81]. Interestingly, HMGB1 release by dying cells during radiotherapy or chemotherapy stimulates tumour cell proliferation by interacting with RAGE and potentiating the ERK/ p38 signalling pathway [82]. In a model of epidermal growth factor receptor‐targeted diphtheria toxin killing, dying cells that induced autophagy released HMGB1, whereas in cells where autophagy was blocked, HMGB1 was retained [83]. Autophagy‐dependent effects on DAMP secretion may be linked to the role the autophagy machinery plays in cellular secretion. Recently, it has been identified that the LC3 conjugation machinery is important for extracellular vesicle cargo loading and secretion, particularly the secretion of RNA‐binding proteins and small noncoding RNAs [84]. Overall, it is clear that DAMP secretion is modulated by autophagy and can potentiate autophagy; however, different DAMPs can have differential effects on tumour growth and tumour clearance.

### 4.3 Autophagy and TLR signalling

Various components of the TLR pathways can stimulate autophagy. For example, MYD88 and TRIF promote autophagosome formation [76]. In a lung cancer model, TLR4 and TLR3 activation caused an induction of autophagy, which enhanced cytokine production and increased the migration and invasion of lung cancer cells [85]. HCC carcinogenesis was increased in TLR2 knockout mice treated with diethylnitrosamine, a potent liver carcinogen [86]. This was linked with a decreased immune response, increased senescence and decreased autophagic flux in liver tissue. It would be interesting to dissect whether TLR2 stimulation could alter autophagic flux in hepatocytes in the context of HCC.

Downstream components of TLR signalling are regulated by autophagy. Indicatively, selective autophagy‐related receptors SQSTM1 (p62) and TAX1BP1 have been shown to regulate TRIF turnover, and loss of the autophagy‐related gene ATG16l1 caused accumulation of TRIF and downstream signalling in macrophages [87]. In this context, human macrophages with the ATG16l1 variant T300A produced more IFN‐β and IL‐1β, demonstrating that autophagy components regulate TRIF to regulate inflammation and innate immunity [87]. It would be interesting to determine whether this regulation of TRIF is lost in cancer cells, where autophagy has been compromised, as this could result in increased inflammation, which subsequently enhances tumorigenesis.

It is clear that autophagy induction can be regulated by PRR signalling as a host defence mechanism, to limit inflammation and aid in the removal of intracellular pathogens. During tumorigenesis, autophagy can regulate DAMP release and components of PRR signalling pathways to modulate cytokine expression and tumorigenic properties of cells. However, autophagy downstream of PRR activation can both aid in the elimination of precancerous cells and enhance tumour phenotypes during tumorigenesis. Conversely, inhibition of autophagy during tumorigenesis can increase the production of inflammatory cytokines to stimulate other aspects of tumorigenesis such as angiogenesis and activation of stromal cells. Therefore, targeting components of the inflammatory pathway to regulate autophagy and vice versa during tumorigenesis may have varying outcomes.

### 4.4 Autophagy cross‐talk with NLR activation

There are various examples of NLR activation by PAMPs and DAMPs inducing autophagy as a feedback loop to ensure a controlled inflammatory response [76]. However, there is limited research on how this feedback loop functions in tumorigenesis. It would be interesting to determine whether different members of the NLR family contribute to autophagy induction in tumour cells and supporting stromal cells, and whether this induction is pro‐ or antitumorigenic. Autophagy also regulates the inflammasome, which can be stimulated by NLRs to cause the secretion of IL‐1β and IL‐18. Mitophagy, a selective form of autophagy that removes damaged mitochondria, inhibits IL‐1β and IL‐18 production by decreasing the build‐up of mitochondrial DAMPs such as ROS and mtDNA [94, 95]. The protein encoded by the UV radiation resistance‐associated gene (UVRAG) has recently been identified as a tumour suppressor that can activate the Beclin1‐PI3KC3 complex to promote autophagy [96]. Mice that express the truncated form of UVRAG, which is observed in cancer, were deficient in starvation‐ and LPS‐induced autophagy, and enhanced inflammation in a colitis‐associated cancer model due to NLRP3 inflammasome activation [96]. These mice also spontaneously developed more tumours in comparison with wild‐type mice, suggesting that the control of the inflammasome by autophagy is important for tumour initiation and tumour progression.

### 4.5 Autophagy regulates the cGAS‐STING pathway

In addition to modulating DAMP expression in cancer, autophagy also feeds back into PRR recognition of DAMPs and PAMPs and downstream inflammatory pathways. Autophagy proteins contribute to the sensing of cytosolic DNA through interactions with the cGAS‐STING pathway. STING seems to be regulated by a noncanonical autophagy pathway, as ATG7 deletion has no effect on STING function, whereas loss of ATG9L1 does [88]. This could be due to the different functions of ATG7 and ATG9L1 in the autophagy pathway, in which ATG9L1 plays a role in the organisation of pre‐autophagosomal structure/ phagophore assembly sites, whilst ATG7 functions as an E1‐like activating enzyme for ATG12 and the ATG8 family [89]. STING can also activate noncanonical autophagy that is dependent on ATG5, which results in degradation of STING following TBK1 activation [90]. The mechanism of autophagy activation by STING has been expanded, whereby cGAMP binding causes STING to translocate to the endoplasmic reticulum–Golgi intermediate compartment. Here, it acts as a membrane source for LC3 lipidation, an event critical for autophagosome formation [91]. The LC3 lipidation induced by cGAMP is dependent on WIPI2 and ATG5, and is important for DNA clearance in the cytosol, an event that is independent from TBK1 and interferon induction [91].

Autophagy activation by STING occurs during replicative crisis, where precancerous cells with perturbed cell cycle checkpoints can be eliminated [92]. The activation of autophagy is needed for cell death induction during replicative crisis and therefore must be inhibited for cancer to be initiated. In ATG5‐ and ATG7‐depleted cells, cytosolic DNA accumulates causing the activation of STING, STAT1 and associated ISG15 expression [93]. This STAT1‐ISG15 axis promotes migration, invasion and proliferation of cells, suggesting that inhibition of autophagy can promote tumour‐associated phenotypes through STING activation [93]. Thus, whilst STING‐induced autophagy may inhibit the development of precancerous cells, autophagy inhibition may be utilised during tumorigenesis to activate STING and promote tumour phenotypes.

#### 4.5.1 Immunogenic cell death

Tumour antigens must be released into the TME to activate myeloid cells and produce an effective antitumour innate and adaptive immune response. This is achieved through various cell death pathways that cause the release of tumour antigens and DAMPs into the extracellular space. Autophagy interacts with cell death pathways such as apoptosis and necroptosis and can therefore influence the release of tumour antigens and DAMPs into the TME. In mouse prostate cells deficient in MAP3K7, RIPK1 and the necrosome are recruited to the autophagosome membrane to potentiate TRAIL‐induced necroptosis [97]. Inhibition of this complex formation on the autophagosome caused apoptosis induction in response to TRAIL. In macrophages lacking ATG16l1, insoluble forms of RIPK1 and RIPK3 accumulated and necroptosis induction by TNF and TLR ligands was enhanced [98]. Furthermore, autophagy inhibition enhances necroptosis induction in K562 cells in response to the tetrahydrobenzimidazole derivative TMQ0153 [99]. This suggests that the ability of autophagy to promote necroptosis may be cell type‐dependent and stimulus‐dependent. Induction of necroptosis *in vivo* via RIPK3 gene delivery can enhance immune checkpoint blockade to increase tumour clearance, demonstrating that necroptosis is an immunogenic form of cell death [100]. Therefore, it is critical to dissect the interaction between autophagy and necroptosis pathways in tumour cells to enhance immunogenic cell death and immunotherapy.

#### 4.5.2 Protection from innate immune cell killing

In addition to influencing cell death pathways in tumour cells, autophagy can influence tumour cell responses to components of the innate immune response and the infiltration of innate immune cells into the TME. As previously mentioned, NK cells can be utilised for effective tumour clearance; however, they can be rendered ineffective by the TME. In melanoma, targeting of autophagy gene *BECN1* increased the infiltration of functional NK cells into tumours [101]. This was due to an increase in expression of CCL5 in autophagy‐defective tumours, a chemokine critical for NK cell infiltration. It would be interesting to investigate in other tumours where NK cell infiltration is poor, whether autophagy inhibition can increase NK cell infiltration and killing of tumour cells. Autophagy can also modulate cancer cell resistance to NK cell cytotoxicity. Breast cancer cells in hypoxic conditions were able to evade NK cell killing via the activation of autophagy and the subsequent degradation of granzyme B *in vitro* [102]. However, in non‐hypoxic conditions restoration of p53 function in breast cancer cells increased their susceptibility to NK cell cytotoxicity via autophagic sequestering of antiapoptotic Bcl‐2 family members, which potentiated granzyme B‐induced apoptosis [103]. It is important to determine the effect of autophagy on tumour cell susceptibility to NK cell cytotoxicity *in vivo* as differences in the TME could alter the ability of autophagy to inhibit/enhance NK cell‐induced apoptosis in tumour cells.

### 4.6 Autophagy and the regulation of innate immune cells in cancer

Autophagy regulates the functioning of many innate immune cells in the context of infection and inflammation; however, there is less research on how the TME alters autophagy pathways in innate immune cells and how this alters cell functioning. Whilst innate immune cells such as myeloid cells and ILCs have the capacity to generate an effective antitumour immune response, these cells have a high degree of functional plasticity and, as such, can become protumorigenic in the TME. By dissecting whether autophagy in these cells enhances antitumorigenic or protumorigenic functions within the TME, it may be possible to enhance the antitumour immune response and immunotherapy.

#### 4.6.1 Autophagy and antigen presentation in DCs

DCs are critical for the generation of an effective adaptive immune response via the presentation of antigens on MHC molecules. Autophagy interacts with antigen processing pathways in DCs and thus can modulate their effectiveness at stimulating the adaptive immune response. In the context of infection, autophagy can direct pathogens into the autophagosome to cause degradation upon autophagosome–lysosome fusion and the promotion of MHC class II antigen presentation [77]. In mice with DCs lacking ATG5 expression, the administration of apoptotic tumour cells elicited a reduced CD4 T‐cell response, suggesting that MHC‐II antigen presentation was impaired [104]. This was linked with an increase in scavenger receptor CD36 and increased lipid accumulation in ATG5‐deficient DCs. More recently, autophagy in PDAC has been shown to selectively target MHC‐I in tumour cells, causing decreased surface expression [105]. Blocking autophagy enhanced antitumour T‐cell responses and had a synergistic effect with immune checkpoint blockade [105]. MHC‐I expression is elevated in DCs in the absence of Atg5 and Atg7 due to decreased endocytosis and degradation, demonstrating that autophagy can also degrade MHC‐I in DCs [106]. It would be interesting to determine whether MHC‐I control by autophagy in DCs is through a similar mechanism as the one described in tumour cells by Yamamoto et al (2020), involving the autophagy cargo receptor NRB1.

Interestingly, many components of autophagy pathways have autophagy‐independent functions in myeloid cells. VSP34 is involved in canonical and noncanonical autophagy and has been shown to play a role in MHC‐I and MHC‐II antigen presentation by DCs. In mice where VSP34 was deleted from the DC compartment, MHC‐I and ‐II antigen presentation was increased [107]. However, VSP34‐deficient CD8α ^+^ DCs lost their ability to cross‐present cell death antigens to T cells resulting in increased metastasis in a B16 melanoma challenge model. It would be interesting to compare autophagy regulation of MHC presentation in cDCs, pDCs and MoDCs in cancer settings to determine whether DC subsets are differentially regulated. Whilst autophagy can influence antigen processing and presentation in DCs, utilising therapeutics targeting autophagy in DCs to increase antitumour immune responses may have unexpected effects due to the potential autophagy‐independent effects of autophagy pathway components. Therefore, it is critical to dissect the importance of different autophagy pathway components in DC antigen presentation.

#### 4.6.2 Autophagy and immune tolerance

Another critical aspect of tumour immunity regulated by myeloid cells such as DCs is immune tolerance [108]. TIM‐4 expression on DCs and TAMs has been linked to decreased tumour immunity in response to chemotherapy‐induced tumour cell death [109]. TIM‐4 activated autophagy in these cells through interaction with AMPKa1, which caused degradation of ingested tumour debris, reduced antigen presentation and an increased immune tolerance to chemotherapy [109]. Similar to antigen presentation, components of the autophagy pathway can have autophagy‐independent functions in the context of immune tolerance. Inhibition of LC3‐associated phagocytosis (LAP) in myeloid cells caused a proinflammatory phenotype in TAMs, and increased phagocytosis of dying tumour cells resulting in increased tumour control, suggesting that LAP increases immune tolerance [110]. LAP utilises autophagy components to conjugate LC3 to phagosomal membranes; however, LAP is distinct from autophagy based on differential roles for Rubicon, NOX2 and other autophagy proteins [111]. Due to similar pathway components between LAP and canonical autophagy, studies that examine the role of autophagy in tumorigenesis utilising *in vivo* models that target LC3 conjugation machinery may also be identifying effects based on the inhibition of LAP. Therefore, it is important to dissect the differential roles of LAP and canonical autophagy in the context of tumorigenesis and the innate immune response.

Overall, this shows that some autophagy pathway components can suppress immune responses and contribute to tolerance in the TME. Myeloid‐derived suppressor cells (MDSCs) contribute to immune tolerance in the TME to promote tumour development. MDSCs from patients and mice with melanoma had increased levels of autophagy, and inhibition of autophagy in the myeloid compartment in mice caused MDSCs to have impaired suppressive activity [112]. This was linked with increased surface MHC‐II expression and an increase in antitumour immunity. Therefore, autophagy inhibition in MDSCs could be beneficial for cancer treatment; however, targeting this inhibition to a particular myeloid subset in the TME may be challenging. It is important to determine whether the inhibition of autophagy in MSDCs is beneficial in other *in vivo* cancer models.

#### 4.6.3 Autophagy regulates TAM phenotypes

TAMs can support tumour growth by producing cytokines, chemokines and growth factors, which support metastasis and angiogenesis, and can encourage an ineffective immune response in the TME. Autophagy interacts with phagocytic signalling and inflammatory pathways to alter macrophage differentiation and cytokine secretion in the context of infection [77]; however, less is known regarding the interaction between the TME, autophagy and TAM functioning. In an orthotopic HCC implantation model, a natural compound called baicalin caused TAM reprogramming and blockage of the growth of implanted HCC [113]. This was linked to the degradation of TRAF2, a component of the TNF signalling pathway, via autophagy and the activation of RELB/p52, an NF‐κB dimer, in TAMs. This suggests that targeting autophagy in TAMs could alter their functional properties and encourage them to become antitumorigenic. It is important to determine which components of autophagy can modulate TAM functioning to create novel therapeutic targets. TFEB is a critical regulator of autophagy and lysosome biogenesis and has been linked with TAM phenotype regulation. TFEB expression inhibited inflammatory response induction and transcripts associated with protumorigenic functions in macrophages *in vitro* [114]. In both human breast cancer and mouse orthotopic breast tumours, TAMs had decreased TFEB expression, and in a mouse model where macrophages had a TFEB deficiency, breast tumour growth and metastasis were increased. Overall, this suggests that expression of TFEB could be critical for regulating TAM protumorigenic functions and could be a therapeutic target for breast cancer [114]. It would be interesting to determine whether other types of cancer development are altered in the TFEB macrophage‐deficient mouse model or whether this is a breast cancer‐specific phenomenon.

Ultimately, autophagy interacts with signalling pathways in myeloid cells that can alter their pro‐ or antitumorigenic capacity. Autophagy pathway components can alter antigen presentation by DCs to influence the formation of the antitumour immune response. Both DCs and TAMs contribute to immunological tolerance during tumorigenesis, and autophagy appears to potentiate tolerance, suggesting that targeting of autophagy could challenge the formation of tolerance within the TME and shift the local immune response to become antitumorigenic. Additionally, autophagy can influence macrophage functionality and could be targeted to reprogramme TAMs to enhance antitumorigenic functions. As autophagy pathway components appear to play autophagy‐independent roles in myeloid cells, it is critical to identify novel functions of autophagy components in myeloid cells and determine whether these components are modified in myeloid cells in response to the TME.

#### 4.6.4 Autophagy in innate lymphoid cells

NK cells and ILCs are critical in tumour cell elimination and the enhancement of an effective antitumour immune response in the TME. Autophagy plays a role in NK cell differentiation and memory; however, the way in which autophagy modulates NK cell functioning in the TME is not well characterised. In the context of viral infection, mitophagy was induced in proliferating NK cells to promote their survival, and mTORC1 inhibition enhanced memory NK cell numbers through an ATG3‐dependent mechanism [115]. Memory NK cells are a long‐lived NK cell population, which can mount a recall response, and therefore are an interesting target for cancer immunotherapy. This report suggests that mitophagy and autophagy contribute to NK cell survival and development of memory NK cells. Studying whether mitophagy and autophagy are activated in NK cells during an antitumour immune response and whether this promotes memory NK cells is critical, as some memory NK cells are antitumorigenic [116]. As discussed above, NK cell infiltration into tumours can be altered by autophagy in tumour cells; however, even if NK cells do infiltrate tumours they are often rendered nonfunctional. Investigating whether TME stimuli such as hypoxia and metabolite availability could affect autophagy pathways in NK cells and thus alter their functioning could provide novel targets to reactivate NK cell activity in tumours. Additionally, it is possible that autophagy pathway components, like in myeloid cells, could play autophagy‐independent roles in NK cell signalling pathways.

The role of different ILC subsets in tumorigenesis is still being characterised, and there has been minimal research on how autophagy regulates ILC function. However, as with other immune cells, autophagy pathway components in ILCs could be targeted during immunotherapy to enhance the antitumour immune response or inhibit the protumour immune response. Recently, the role of autophagy in ILC2 function has been assessed in an allergic asthma model in *Rag* ^‐/‐^ *Gc* ^‐/‐ ^mice where autophagy‐deficient ILC2s were adoptively transferred [117]. *Atg5* deletion from ILC2s caused a decrease in cytokine secretion and higher levels of apoptosis. This was attributed to metabolic reprogramming in which glycolysis was utilised rather than fatty acid oxidation. As ILC2s appear to have both pro‐ and antitumorigenic effects depending on the tumour model, it would be interesting to determine whether autophagy plays a role in dictating the function of ILC2s within the TME.

## 5 Concluding remarks

Both autophagy and the innate immune response play important roles at tumour initiation, growth and metastasis. Autophagy can help prevent tumour initiation by limiting the production and build‐up of damaged proteins and organelles, which can contribute to a mutagenic environment, whilst inflammation can perpetuate these mutagens to enhance tumour initiation. During tumour growth, components of the innate immune response are manipulated in both the tumour cells and the TME cells to limit an effective immune response and increase tumour growth and metastasis. Discovering factors that cause tumour cells to become nonimmunogenic and innate immune cells to become protumorigenic could identify novel targets to enhance the antitumour immune response and immunotherapy. It is clear that autophagy and the innate immune response are inextricably connected, with autophagy being induced by innate signalling pathways and acting as an essential regulator of inflammation and innate immune cells. Targeting autophagy components in tumour cells could increase tumour immunogenicity due to crosstalk with cell death and DAMP release pathways, whilst targeting autophagy in innate immune cells, such as myeloid cells and ILCs, could enhance antitumorigenic functions. Targeting autophagy in specific cell types in solid tumours could be challenging; therefore, increasing our understanding of the non‐autophagic roles of the autophagy pathway components may lead to more specific and effective targets for therapeutics and immunotherapy.

## Conflict of interest

The authors have no financial conflicts of interest to declare. KMR is Co‐Editor‐in‐Chief of *Molecular Oncology*, but he was not involved in the review of this manuscript.

## Author contributions

CG and KMR wrote the manuscript.
